# Transcatheter arterial chemoembolization combined with apatinib and camrelizumab for unresectable advanced gastric or gastroesophageal junction cancer: a single-arm, single-center, retrospective study

**DOI:** 10.3389/fonc.2023.1143578

**Published:** 2023-09-06

**Authors:** Kunpeng Wu, Yahua Li, Zongming Li, Zihe Zhou, Xiaoyong Ge, Yifan Li, Xinwei Han, Peng Chen, Kewei Ren

**Affiliations:** ^1^ Department of Interventional Radiology, The First Affiliated Hospital of Zhengzhou University, Zhengzhou, Henan, China; ^2^ Interventional Institute of Zhengzhou University, Zhengzhou, Henan, China; ^3^ Interventional Treatment and Clinical Research Center of Henan Province, Zhengzhou, Henan, China; ^4^ Department of Gastrointestinal Surgery, The First Affiliated Hospital of Zhengzhou University, Zhengzhou, Henan, China

**Keywords:** TACE, targeted therapy, immunotherapy, gastric or gastroesophageal junction cancer, comprehensive therapy

## Abstract

**Purpose:**

This study aims to investigate the efficacy and safety of transcatheter arterial chemoembolization (TACE) combined with Apatinib and Camrelizumab for treating unresectable advanced gastric or gastroesophageal junction (G/GEJ) cancer.

**Material and methods:**

In this study, data of patients with unresectable advanced G/GEJ cancer who received TACE combined with Apatinib and Camrelizumab from August 2018 to December 2021 was evaluated. After TACE, patients were given intravenous Camrelizumab 200mg every three weeks and oral apatinib 250mg/day for treatment. The primary endpoint was overall survival (OS), and the secondary endpoints were objective response rate (ORR), disease control rate (DCR), and adverse events (AEs).

**Results:**

A total of 49 patients were enrolled in this study. The median follow-up time was 14.0 months, and the median OS was 20.0 months (95% CI = 13.6-26.4). Two patients (4.08%) achieved complete remission, 28 patients (57.14%) achieved partial remission, 18 patients (36.73%) had stable disease, and 1 patient (2.04%) had disease progression. The ORR was 61.22%, and the DCR was 97.96%. Multivariate Cox regression analysis indicated that age (HR 4.74, 95% CI = 1.674-13.440, P=0.003) and multiple distant metastases (HR 20.916, 95% CI = 4.094-106.808, P = 0.001) were independent risk factors for OS. Most AEs were classified as grade 1-2, the most common being RCCEP (69.39%). There were 5 cases of grade 3-4 adverse events (10.20%). No patients discontinued or reduced the treatment dose due to AEs, and all patients received symptomatic treatment.

**Conclusion:**

TACE combined with Apatinib and Camrelizumab is a safe and effective therapeutic option for patients with unresectable advanced G/GEJ cancer, which can significantly improve the median OS and ORR of patients. And the adverse events (AEs) are tolerable and manageable.

## 1 Introduction

Gastric cancer is the fifth most common cancer and the fourth leading cause of cancer-related death globally (1). However, early diagnosis of gastric cancer is less than 10%, and most patients are already in the late stage when they are diagnosed. Palliative treatment is mainly adopted for late-stage gastric cancer, and the overall prognosis is poor (2–4).

For late-stage gastric cancer, neoadjuvant therapy is one of the most essential parts of treatment, neoadjuvant therapy can shrink the tumor, thus enabling radical resection and prolonging the patient’s survival (5, 6). Although new adjuvant chemotherapy could effectively control the development of tumors, some patients suffer severe side effects. TACE has emerged as a standard treatment for liver cancer and a new therapeutic option for the treatment of gastric or gastroesophageal junction (G/GEJ) cancer. There were relatively few reports on TACE for the treatment of gastric cancer (7). Some case reports have demonstrated the efficacy and safety of transcatheter arterial therapy in late-stage gastric cancer, indicating that TACE may have more prospects in the treatment of gastric cancer (8, 9). TACE can increase the concentration of localized chemotherapy drugs and reduce adverse reactions (10). The principle of TACE treatment for gastric cancer is to inject chemotherapeutic drugs and embolic agents into the tumor blood supply arteries through vascular super-selective technique, thus increasing the local blood concentration, which not only enhances the ability to kill tumor cells, but also reduces the systemic toxic side effects of chemotherapeutic drugs. In addition, embolic agents can further cut off the tumor trophoblastic arteries and slow down the invasion and metastasis of tumor cells, thus controlling tumor progression (11).

Oxaliplatin is a commonly used chemotherapeutic drug in the treatment of gastric cancer, which can induce immunogenic cell death (12). TACE can effectively kill malignant tumor cells or tissues by localized chemotherapy and vascular occlusion, thus exposing tumor antigens and promoting tumor immunization. However, the antitumor effect of TACE is not last. After the tumor-feeding artery is blocked, the tumor microenvironment is in a hypoxic and ischemic state, which induces the expression of vascular endothelial growth factor (VEGF) and promotes tumor vascularization, ultimately leading to tumor recurrence and metastasis (13, 14).

Immune checkpoint inhibitors (ICIs) can activate systemic antitumor immune response by blocking programmed cell death protein 1 (PD-1) signal transduction (15). Camrelizumab (SHR-120) is a high-affinity and completely humanized monoclonal antibody against PD-1 (16). In the first-line setting, ICIs in combination with chemotherapy for advanced G/GEJ cancer significantly prolong OS and PFS in patients (17–19). However, the effect of single immunotherapy is not satisfactory compared with conventional treatments. Abnormal angiogenesis is considered a fundamental cause of solid tumor immune escape (20). Therefore, ICIs combined with anti-angiogenic drugs for the treatment of gastric cancer have attracted a lot of attention (21, 22). Apatinib is a small molecule tyrosine kinase inhibitor that binds and strongly inhibits VEGFR2 and reduces tumor angiogenesis (23). It has been approved in China as a three-line drug for the treatment of advanced gastric cancer (24, 25). Current studies suggest that VEGF has immunosuppressive effects and that anti-angiogenic drugs targeting VEGF-VEGFR can increase T cell activity and promote infiltration and activation of CD8+ T cells in the tumor microenvironment, thus acting synergistically with ICIs (26). In some studies, apatinib have been shown to reprogram TME, reverse immune-suppressive to inflamed state, and enhance the efficacy of ICIs (18, 27). In addition, apatinib can effectively inhibit reactive skin microvascular endothelial proliferation induced by camrelizumab (28). Therefore, ICI combined with anti-angiogenesis drugs can improve the effectiveness of immunotherapy, reduce the adverse effects of immunotherapy and improve the prognosis of patients. As TACE can induce immunogenic cell death, TACE combined with immunotherapy can also enhance efficacy (29).

Apatinib combined with camrelizumab can improve the clinical benefit of patients with unresectable advanced G/GEJ cancer (30). However, there have been no reports on TACE combined with apatinib and camrelizumab for the treatment of advanced gastric cancer patients. In this single-arm, single-center, retrospective study, we aim to explore the safety and effectiveness of TACE combined with apatinib and camrelizumab for the treatment of unresectable advanced G/GEJ cancer.

## 2 Materials and methods

### 2.1 Patients

This retrospective study was approved by the Ethical Review Board of our hospital and was conducted in accordance with the Declaration of Helsinki. Patients with unresectable stage G/GEJ cancer who received TACE combined with Apatinib (Jiangsu Heng Rui Medicine Co., Ltd., Jiangsu, China) and Camrelizumab (Jiangsu Heng Rui Medicine Co., Ltd., Jiangsu, China) treatment from August 2018 to December 2021 were included. The main inclusion criteria were as follows: 1. Age ≥ 18 years; 2. Pathological and/or cytological diagnosis of gastric adenocarcinoma or adenocarcinoma at the gastroesophageal junction; 3. At least one measurable lesion; 4. Eastern Cooperative Oncology Group (ECOG) score: 0 or 1; 5. Estimated survival ≥ 6 months; 6. Normal renal function or corrected by appropriate treatment; 7. Patients refused to receive surgery and had not received any interventional therapy in the past. Exclusion criteria included: 1. Patients with severe coagulation dysfunction, renal insufficiency, or cardiopulmonary dysfunction that cannot be corrected; 2. Patients who are allergic to contrast agents and chemotherapy drugs (such as oxaliplatin); 3. Pregnant or lactating women; 4. Severe infection; 5. Insufficient data (For example, incomplete follow-up data, loss of image data, etc.).

### 2.2 Treatment protocol

**Figure 1:**
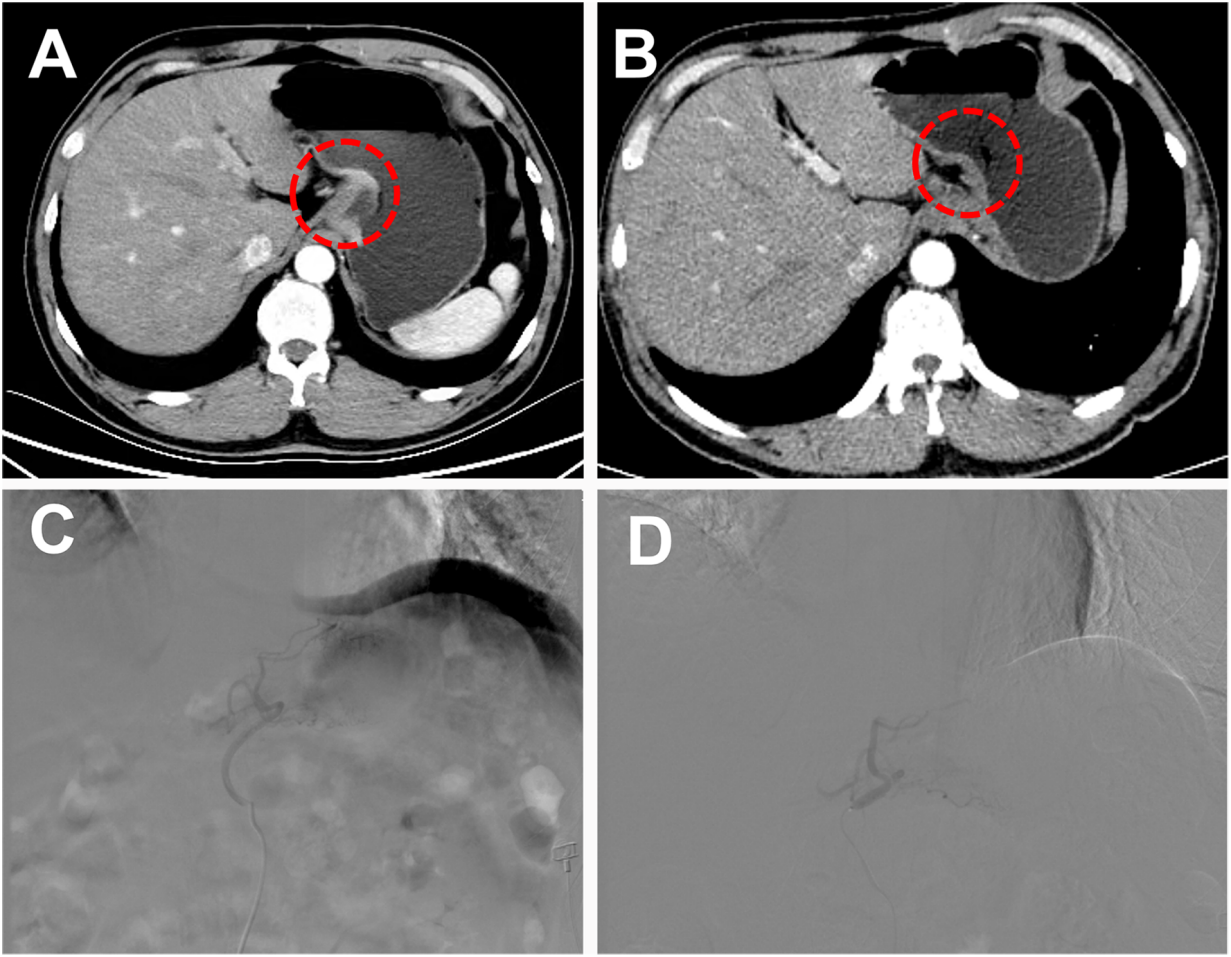
A 51-year-old male patient with low-grade adenocarcinoma at the gastroesophageal junction was offered a curative surgical opportunity after TACE combined with targeted drugs and immunotherapy. **(A)** Abdominal Computer Tomography (CT) showed a tumor at the gastroesophageal junction during the arterial phase before combined treatment. **(B)** After 4 months of combined treatment, the patient’s abdominal CT arterial phase enhancement of the original tumor disappeared. **(C)** Under the guidance of digital subtraction angiography (DSA), the tip of the 5-F catheter with guidewire was introduced to the aorta, and select the left gastric artery to perform angiography and observed the blush of the tumor. **(D)** The operator slowly injected the iodized oil-oxaliplatin emulsion through the microcatheter until the tumor blush disappeared.

Routine disinfection and local anesthesia were performed at the puncture site with 5 ml of 2% lidocaine. A 5-French (F) sheath was inserted into the femoral artery using the modified Seldinger technique. Under the guidance of digital subtraction angiography (DSA), the tip of the 5-French (F) catheter (COOK, USA) with guidewire was introduced to the aorta and selected the left gastric artery to perform angiography. After observing the blush of the tumor, a 2.7-French (F) microcatheter (Terumo, Tokyo, Japan) was super-selected into the tumor-feeding artery for embolization. The embolization technique included the following: iodized oil-oxaliplatin emulsion, an oil-in-water type chemical embolic agent prepared by mixing oxaliplatin with iodized oil. The operator slowly injected the iodized oil-oxaliplatin emulsion through the microcatheter until the tumor blush disappeared (**Figure 1**). The TACE was ended. One week after TACE, the patient was given 200mg of camrelizumab monoclonal antibody intravenously every three weeks and 250mg of apatinib orally per day for treatment. The patient could undergo multiple TACE as needed, and apatinib was suspended 3 days before the subsequent TACE surgery.

### 2.3 Study endpoints

The primary endpoint of this study was overall survival (OS). OS was defined as the time from receiving the first TACE treatment to death due to any reason. The secondary endpoints of this study included objective response rate (ORR), disease control rate (DCR), and treatment-related adverse events (AEs). Tumor responses were assessed by two experienced radiologists using the Response Evaluation Criteria in Solid Tumors (RECIST, version 1.1) (31), including complete response (CR), partial response (PR), disease stability (SD), and progressive disease (PD). ORR was defined as CR + PR, and DCR was defined as CR + PR + SD. Adverse events were assessed according to the Common Terminology Criteria for Adverse Events (CTCAE, version 4.03).

### 2.4 Statistical methods

All data were analyzed using SPSS 21.0 (IBM Corp., Armonk, NY, USA). All continuous variables were reported as means ± standard deviation (SD) or median (min-max), and categorical variables were presented as numbers (percentages). Student’s t-test was used to compare continuous data, and Chi-square or Fisher’s exact test was used to compare categorical data. Survival curves were estimated by the Kaplan-Meier method, and the median OS was calculated from it. Multivariate Cox regression analysis was used to predict factors affecting OS. P-value of <0.05 was considered significant.

## 3 Results

### 3.1 Patient demographics

A total of 49 advanced G/GEJ cancer patients participated in this study from August 2018 to December 2021. The baseline characteristics of the patients are shown in **Table 1**. The median age was 67 years (range 33-89 years), 46 (93.88%) were male, 3 (6.12%) were primary tumors in the stomach, and 46 (93.88%) were at the gastroesophageal junction. 34 (69.38%) patients had distant metastases, 18 patients underwent only one TACE procedure, and 31 patients underwent 2-4 TACE procedures. 13 patients achieved R0 resection after downstaging with TACE combined with apatinib and camrelizumab.

**Table 1:** Patient characteristics at baseline.

Characteristics	All patients enrolled (n = 49)
Age(years), media(range)	67 (33-89)
Age group(years) (%)	
<65	18 (36.73)
≥65	31 (63.27)
Sex (%)	
Male	46 (93.88)
Female	3 (6.12)
ECGO PS (%)	
0	25 (51.02)
1	24 (48.98)
Location of primary tumor (%)	
Gastric	3 (6.12)
Gastroesophageal junction	46 (93.88)
Laboratory parameters	
RBC (109/L, mean ± SD)	3.91 ± 0.64
WBC (1012/L, mean ± SD)	5.56 ± 3.33
Hb (g/L, mean ± SD)	112.96 ± 25.38
Comorbidities (%)	
Hypertension	5 (10.20)
Diabetes mellitus	2 (4.08)
Coronary heart disease	3 (6.12)
Organs with metastases (%)	
1	15 (30.61%)
≥2	34 (69.38%)
Number of TACE (%)	
<2	18 (36.73)
≥2	31 (63.27)
Radical operation (%)	
Yes	13 (26.53)
No	36 (73.47)
Data are median (range) or N (%).	

ECOG PS, Eastern Cooperative Oncology Group Performance Status; RBC, red blood cell; Hb, hemoglobin; WBC, white blood cell; TACE, transcatheter arterial chemoembolization.

### 3.2 Efficacy

**Figure 2:**
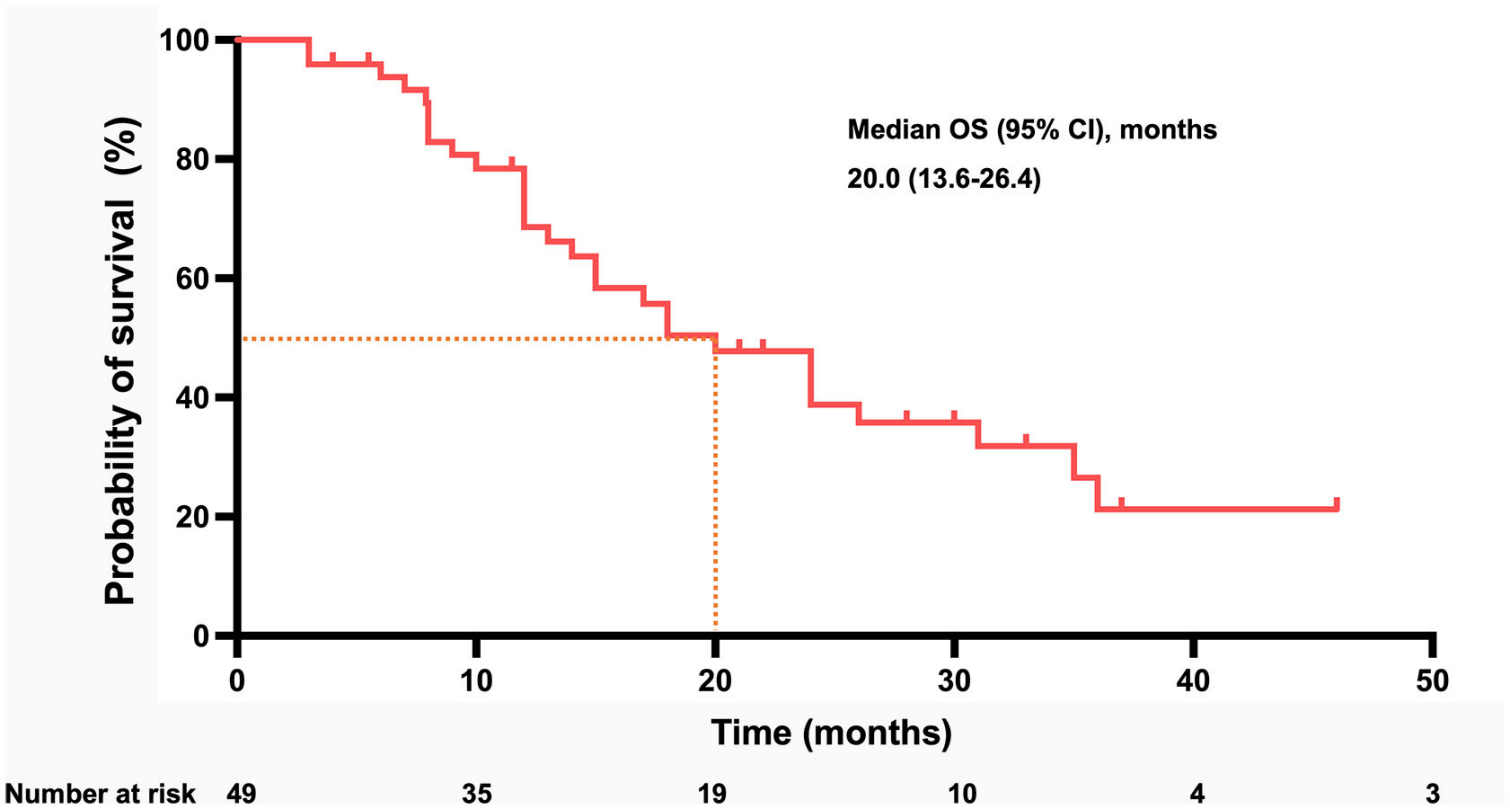
Kaplan–Meier curve for OS in all patients of cohort. (OS, overall survival; CI, confidence interval).

**Figure 3:**
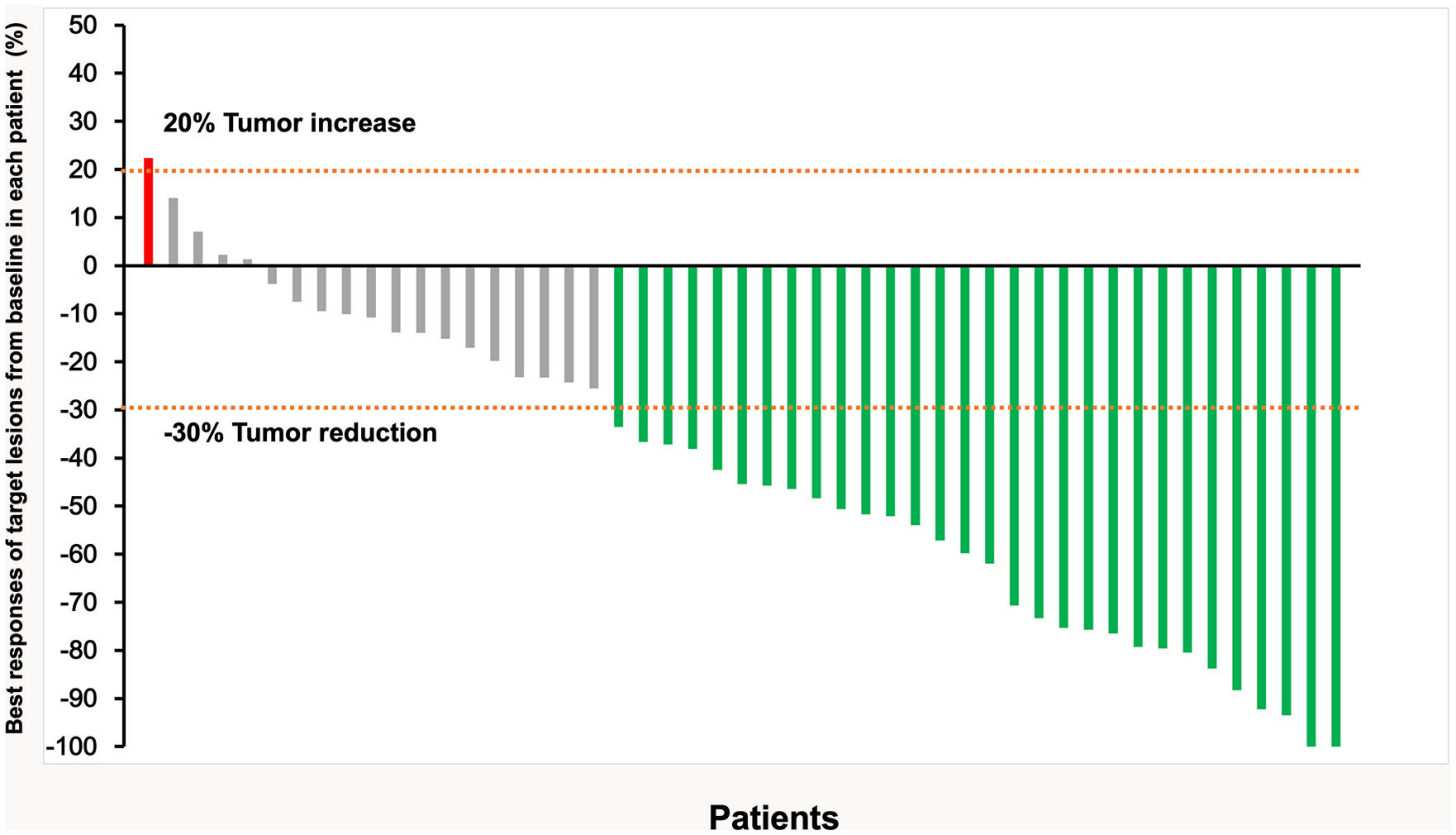
Responses in all 49 patients were assessed according to RECIST v1.1, the best responses of target lesions from baseline in each patient (Each vertical line represents an individual patient).

By the time of follow-up (May 2022), the median follow-up was 14.0 months, and the median OS was 20.0 months (95% CI =13.6-26.4) (**Figure 2**). Univariate Cox regression risk proportional model (**Table 2**) showed that age (HR 4.445, 95% CI =1.865-10.592, P <0.001), ECGO score (HR 2.189, 95% CI =1.032-4.651, P= 0.041), comorbidities (HR 2.686, 95% CI =1.044-6.910, P=0.040), TACE number (HR 2.184, 95% CI= 1.006-4.741, P=0.048) and distant metastasis (HR 12.647, 95%CI = 3.552-45.036, P= 0.001) were risk factors for OS. Multivariate Cox regression (**Table 2**) showed that the independent risk factors for OS were age (HR 4.74, 95% CI=1.674-13.440, P= 0.003) and distant metastasis (HR 20.916, 95% CI=4.094-106.808, P=0.001). Tumor response in all patients was assessed according to RECIST 1.1 criteria (**Table 3**), with 2 patients (4.08%) achieving a complete response, 28 patients (57.14%) achieving a partial response, 18 patients (36.73%) being stable, and 1 patient (2.04%) progressing. The objective remission rate was 61.22%, and the disease control rate was 97.96% (**Figure 3**).

**Table 2:** Univariate Cox proportional hazards regression model analysis for OS.

	Univariate analysis			Multivariate analysis		
	HR	95% CI	P-value	HR	95% CI	P-value
Age group(years) (<65 vs ≥65)	4.445	1.865-10.592	0.001	4.743	1.674-13.440	0.003
Sex (Male vs. Female)	1.047	0.247-4.448	0.95			
ECGO PS	12.066	3.890-37.430	0.001	1.919	0.783-4.704	0.154
RBC	1.11	0.531-2.321	0.781			
WBC	0.935	0.448-1.948	0.857			
Hb	1.774	0.800-3.932	0.158			
Comorbidities	2.686	1.044-6.910	0.04	0.806	0.255-2.545	0.713
Number of TACE (<2 vs ≥2)	2.184	1.006-4.741	0.048	0.995	0.399-2.484	0.992
Organs with metastases	12.647	3.552-45.036	0.001	20.916	4.094-106.808	0.001

ECOG PS, Eastern Cooperative Oncology Group Performance Status; RBC, red blood cell; Hb, hemoglobin; WBC, white blood cell; TACE, transcatheter arterial chemoembolization.

**Table 3:** Summary of tumor response based on RECIST 1.1.

Tumor response	All patients (n=49, %)	
CR	2	4.08%
PR	28	57.14%
SD	18	36.73%
PD	1	2.04%
ORR(CR+PR)	30	61.22%
DCR(CP+PR+SD)	48	97.96%

RECIST, Response Evaluation Criteria in Solid Tumors; CR, complete response; PR, partial response; SD, stable disease; PD, progressive disease; ORR, objective response rate; DCR, disease control rate.

### 3.3 Safety

No TACE related severe complications happened. No deaths related to treatment were observed (**Table 4**). The most common adverse events associated with apatinib and carfilzomib included skin reactions of the hands and feet (30.61%), diarrhea (26.53%), hypertension (20.41%), and proteinuria (20.41%). Typical adverse events associated with TACE included fever (55.10%), pain (51.02%), nausea and vomiting (48.98%), diarrhea (26.53%), and abdominal distension (16.33%). According to CTCAE 4.03, most AEs were classified as Grade 1-2, with RCCEPs being the most common (69.39%) and Grade 3-4 adverse events 5 cases (10.20%). No patients discontinued or reduced due to adverse events, and all patients were corrected after symptomatic treatment.

**Table 4:** Treatment-related adverse events.

n (%)	n=49	
	All grade	Grade≥3
Fever	27 (55.10%)	1 (2.04%)
Nausea and vomiting	24 (48.98%)	0
Pain	25 (51.02%)	0
Hand-foot skin reaction	15 (30.61%)	0
Hypertension	10 (20.41%)	1 (2.04%)
Fatigue	12 (24.49%)	0
Mouth ulcers	1 (2.04%)	0
Proteinuria	10 (20.41%)	0
Hypothyroidism	2 (4.08%)	0
Hyperthyroidism	5 (10.20%)	0
diarrhea	13 (26.53%)	2 (4.08%)
abdominal distension	8 (16.33%)	0
RCCEP	34 (69.39%)	0
Hemorrhage, upper GI	2 (4.08%)	0
Leukopenia	20 (40.82%)	0
Thrombocytopenia	25 (51.02%)	1 (2.04%)
Anemia	29 (59.18%)	0

RCCEP, reactive cutaneous capillary endothelial proliferation.

## 4 Discussion

In recent years, the treatment of unresectable advanced G/GEJ cancer has developed rapidly. A variety of treatments including targeted drugs, immune checkpoint inhibitors, and TACE have been used for unresectable advanced G/GEJ cancer (32). However, monotherapy has unsatisfactory efficacy, and combination therapy can provide additional clinical benefits for patients. The phase 3 trial of EPOC 1706 reported that lenvatinib plus pembrolizumab had significant anti-tumor activity in first- or second-line treatment of patients with advanced gastric cancer, with an overall response rate of 69% and a median progression-free survival of 7.1 months (27). The study of Peng et al. showed that combined with apatinib, camrelizumab treatment of advanced or metastatic G/GEJ adenocarcinoma had an ORR of 58.3%, which was superior to the standard first-line chemotherapy regimens reported previously, showing encouraging anti-tumor activity and controllable toxicity (18).

TACE can effectively kill tumor cells or tissues through regional chemotherapy and vascular occlusion (33). As a potential treatment, TACE has been included in the 2022 Chinese guideline for the diagnosis and treatment of gastric cancer and is used for palliative treatment or adjuvant treatment of advanced gastric cancer and unresectable gastric cancer (34). The study of Li et al. showed that TACE treatment for advanced gastric cancer was safe and effective and could reduce tumor size and improve the quality of life of patients (7). In a prospective, single-arm, single-center clinical study, Su et al. confirmed that TACE combined with apatinib and S-1 could significantly improve the objective response rate (ORR 94.7%) and R0 resection rate (63.0%) of patients (35). In addition, TACE and apatinib can also promote tumor immunity and improve the efficacy of ICI in various ways. Still, there is no report of the combined use of the three treatments.

This single-arm, single-center, retrospective study showed that TACE combined with apatinib and carrelizumab in the treatment of unresectable advanced G/GEJ cancer, the median overall survival was 20.0 months (95% CI 13.6-26.4), the objective response rate was 61.22%, and the disease control rate was 97.96%. Through Cox multivariate regression analysis, we found that age and multiple distant metastases were independent risk factors affecting the prognosis of patients. In the REGONIVO trial, nivolumab combined with regorafenib for advanced GC patients, the median PFS was 5.6 months, and the median OS was 12.3 months, ORR was 44% (36); in the second-line treatment of advanced G/GEJ cancer, a patient with camrelizumab plus apatinib plus S-1, ORR was 29.2%, median PFS was 6.5 months (37); in untreated advanced GC patients, camrelizumab plus CAPOX for 4-6 cycles after camrelizumab induced no disease progression, the median OS was 14.9 months (95% CI, 13.0-18.6), and ORR was 58.3% (28/48; 95% CI, 43.2-72.4) (18). In a single-armed, phase II, exploratory trial (NCT03878472), Complete and major pathological response rates are 15.8% and 26.3% for cariolizumab in combination with apatinib and chemotherapy for locally advanced gastric cancer, respectively (38). Our results are better than those of the above studies, which may indirectly prove that TACE combined with anti-angiogenesis drugs and immune checkpoint inhibitors has the advantage of treating unresectable advanced G/GEJ cancer. In fact, TACE combined with apatinib and camrelizumab for the treatment of malignant tumors has a sufficient theoretical basis. Firstly, TACE can deliver high-concentration chemotherapeutic drugs directly to the tumor site and slowly release and kill tumor cells and tissues. In addition, embolization materials can also block the nutrient arteries of the tumor, causing tumor ischemia and hypoxia necrosis, releasing tumor antigens, increasing the expression of PD-1/PD-L1, and enhancing the tumor’s response to ICI drugs. Secondly, apatinib can highly selectively bind and strongly inhibit VEGFR2, reduce tumor angiogenesis, further inhibit recurrence and metastasis caused by tumor hypoxia after TACE surgery, and apatinib can also regulate immune suppression through anti-angiogenesis, enhance the antitumor activity of ICI drugs, and effectively reduce the RCCEP induced by camrelizumab, all of which can improve the clinical benefit of patients (12–14, 26, 28, 29).

This study showed that TACE combined with apatinib and camrelizumab for the treatment of advanced unresectable G/GEJ cancer has excellent safety, most AE were classified as grade 1-2, the most common of which was RCCEP (69.39%), and grade 3, 4 AEs were 5 cases (10.20%). There was no TACE-related digestive tract bleeding and perforation, which was similar to previous reports, indicating that TACE still has high safety when used in hollow organs (7, 35).

## 5 Limitation

This study has several limitations. First, this study is a single-arm,single-center, retrospective study with a small population, which may reduce the study’s statistical power. Secondly, some patients did not receive long-term regular treatment with apatinib plus camrelizumab after TACE treatment because of their family’s economic difficulties and high drug prices. Thirdly, in addition to 13 patients who completed radical surgery, some patients still have the opportunity to receive radical surgery, they give up surgery due to personal reasons or economic conditions, which leads to the limited reference significance of the radical surgery rate in this study. Finally, the follow-up time is relatively short, and some patients have not observed the endpoint events. Therefore, further prospective studies or larger sample size randomized controlled trials are needed to verify these results.

## 6 Conclusion

In conclusion, this study demonstrates that the combination of TACE with Apatinib and Camrelizumab is a safe and effective treatment for advanced unresectable G/GEJ cancer. It greatly enhances patients’ median OS and ORR, while the AEs are manageable and well-tolerated. This combination therapy offers an effective neoadjuvant treatment option for patients with unresectable G/GEJ cancer, potentially enabling surgical intervention. In the future, further research can be conducted to delve into the specific molecular mechanisms of TACE combined with Apatinib and Camrelizumab treatment. This will help generate more rational and reliable data, as well as theoretical support for clinical practice.

## Data availability statement

The original contributions presented in the study are included in the article/supplementary material. Further inquiries can be directed to the corresponding authors.

## Ethics statement

The studies involving humans were approved by Ethics Review Committee of the First Affiliated Hospital of Zhengzhou University. The studies were conducted in accordance with the local legislation and institutional requirements. Written informed consent for participation was not required from the participants or the participants’ legal guardians/next of kin in accordance with the national legislation and institutional requirements. Written informed consent was obtained from the individual(s) for the publication of any potentially identifiable images or data included in this article.

## Author contributions

KW, YHL, ZL, ZZ, XG, YFL made data analysis and interpretation. KW, YHL, XH, PC and KR were major contributors. All authors made a substantial contribution to researching data, discussion of content, reviewing and editing the manuscript before submission. All authors read and approved the final manuscript.
